# Diseases of the Stomach and Intestines

**Published:** 1886-11

**Authors:** 


Diseases of the Stomach and Intestines. A Manual of Clinical Therapeutics for the Student and Practitioner. By Prof. Dujardin-Beaumetz, physician to the Cochin Hospital; member of the Academy of Medicine and of the Council of Hygeine and Salubrity of the Seine; Editor-in-Chief of the *Bulletin Generel de Therepeutique*, etc. Translated from the fourth French edition by E. P. Hurd, M. D., President of the Essex North District Medical Society; member of the Massachusetts Medical Society and of the Climatological Society; one of the physicians to the Anna Jaques Hospital, Newburyport, Mass., with illustrations and one chromo-lithograph. New York: William Wood & Co. 1886.

The well-established character of the past contributions of the author of this work is supported by an array of his productions in the introduction of the translator, which might have been appropriately substituted with a more definite outline of the present undertaking, in regard to which the reader is supposed to be chiefly interested. In the liberty taken in the abridgement and condensation of the notes, he would have been pardoned in leaving out such matters as the formulæ of comments for King Louis XIV., and details touching the prosecution of Bourgeois by Etiennette for payment of her injections for two years. Had the author forgotten the lore of past centuries, and made fewer references to the fathers of medicine, it would not have detracted from this valuable contribution to the elucidation of the diseases of the stomach and intestines.

After discounting these slight blemishes, and thus clearing away the mist, let us bring into view the important theoretic and practical features of the original investigations herein presented for the guidance of students and practitioners.

The author concurs in the view of Brinton as to the inappropriateness of the term dyspepsia in connection with the diseases of the stomach, yet being understood, he has adopted a division of dyspepsias which is established upon a physiological basis.

The book is devoted to the consideration of primordial alimentary principles, complete and complex aliments, alimentation, regimen, lavage and gavage of the stomach, putrid dyspepsia, acid and pituitous dyspepsia; treatment of atonic dyspepsia and of dilatation of the stomach; treatment of vomiting; treatment of neuroses of the stomach; buccal and intestinal dyspepsia; secondary dyspepsia; dyspepsia of new-born infants; ulcer and cancer of the stomach; the intestine from a therapeutic standpoint; the hygienic treatment of constipation; saline purgatives; saccharine, drastic and mechanical purgatives; treatment of intestinal occlusion; diarrhœa and dysentery; with treatment of hemorrhoids and intestinal worms. It is the work of an expert in this field.

